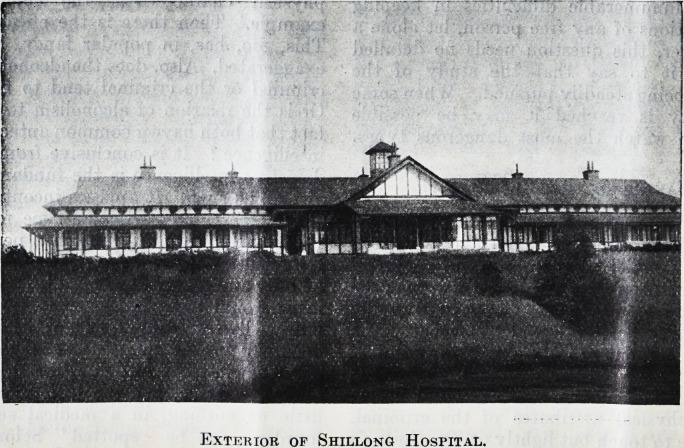# A Mission Hospital in Assam

**Published:** 1924-04

**Authors:** 


### A MISSION HOSPITAL IN ASSAM.

### IN THE KHASI HILLS.

SITUATED on a pine-clad hill on the borders of Shillong is the Khasi Hills Welsh Mission Hospital. The hospital itself, which is designed to accommodate ninety Indian patients, is the outcome of years of devoted labour on the part of the Welsh Mission staff. Since the great earthquake of 1897, when the large Mission Hospital in Chenapoongee was destroyed, there has been no proper Hospital in the Khasi Hills, and the pressing need for greater medical facilities manifested itself so strongly that the Mission staff felt that a new Hospital must be built at any cost.

Part of the Women's Ward in Shillong Hospital.

It will be readily recognised that, in erecting and equipping the Hospital, the Mission has had to undertake a very heavy financial burden. A Special Fund of upwards of £30,000 was raised in Wales to provide an Endowment Fund, from which, it was hoped, the accruing interest, together with outside subscriptions, would furnish an income for the maintenance of the Hospital. Unfortunately, however, the increased cost of materials, the rise in wages, etc., have forced the promoters to encroach sadly upon the capital fund. The Hospital is built on thoroughly modern lines, and equipped with all recent appliances, and is, indeed, a model of what a hospital should be. The main building contains four delightful roomy wards, two surgical and two medical, and eight private wards, where Europeans can go, consulting-rooms with special Eye room, together with a large Dispensing room, Laboratory and Outpatients' dressing rooms. The Operating Theatre is probably as modern as anything in Europe. All the sterilizing is done by electricity, and how those sterilizers shine! This is not because they are out of use, for a great deal of very efficient surgical work is done in the Hospital. The X-Ray room is excellent, too, and contains a thoroughly modern installation.

Entirely separate from the main building is the Segregation Block, which contains four wards for infectious diseases. Special attention has been given to ventilation, and modern baths, etc., have been fitted, so that nothing may be lacking to promote cleanliness. The installation of a Septic Tank, together with admirable sanitary arrangements, will, it is hoped, assist in maintaining healthy conditions. The Hospital and adjoining buildings are lighted throughout with electricity, and it is to be noted that the whole of the electrical installation, including the erection and setting up of engine and power house, has been done by Khasis, as indeed has been the case with the whole of the scheme, with the exception of the sanitary installation. Adequate quarters have been allotted to the staff and a "Serai" for the relatives of the patients has been included. In making a tour of this beautiful Hospital the thing that most strikes the observer is the atmosphere of peace and happiness. One feels that the patients were glad to be there, and an expression of absolute trust was stamped on many faces. The number of Christians in these hills is fairly large,

Exterior of Shillong Hospital.

but the Mission Staff have many opportunities to liberate men from the power of demon worship.

At the present time there are five-and-twenty Khasi Nurses who are splendidly taught by their capable Matron and Medical Officer in Charge. All the nurses are bright and happy and very attentive to the sick. A patient in the private wards declared that it was a treat to be nursed by the Khasi—nothing seems too much trouble for them. The Hospital, like all others, is greatly in need of funds to carry on the splendid work, and any contribution is acceptable and may be sent to the Rev. R. Williams, General Secretary, 16, Falkner Street, Liverpool, or to the Medical Missionary in Charge of the Hospital. Perhaps this article may catch the eye of some nurse who has long felt the call to the mission field and yet has not dared to respond ; if so, let her rest assured that in the Khasi Hills she will have a welcome and ample opportunity for both spiritual and nursing work.

## Figures and Tables

**Figure f1:**
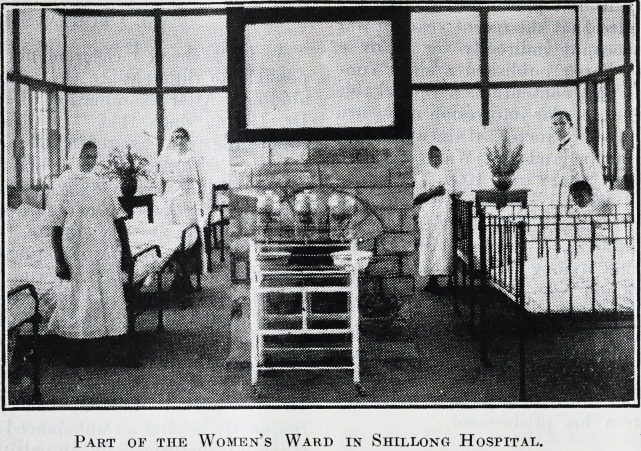


**Figure f2:**